# Depicting Binding-Mediated Translocation of HIV-1 Tat Peptides in Living Cells with Nanoscale Pens of Tat-Conjugated Quantum Dots

**DOI:** 10.3390/s17020315

**Published:** 2017-02-10

**Authors:** Chien Y. Lin, Jung Y. Huang, Leu-Wei Lo

**Affiliations:** 1 The T.K.P. Research Center for Photonics, Chiao Tung University, Hsinchu 300, Taiwan; raw476@gmail.com; 2 Institute of Biomedical Engineering and Nanomedicine, National Health Research Institutes, Zhunan 350, Taiwan; lwlo@nhri.org.tw

**Keywords:** single-particle tracking, cell-penetrating peptides, heparan sulfate proteoglycan, actin filaments, live cell, plasma membrane

## Abstract

Cell-penetrating peptides (CPPs) can translocate across cell membranes, and thus have great potential for the cellular delivery of macromolecular cargoes. However, the mechanism of this cellular uptake process is not yet fully understood. In this study, a time-lapse single-particle light-sheet microscopy technique was implemented to obtain a parallel visualization of the translocating process of individual human immunodeficiency virus 1 (HIV-1) transactivator of transcription (Tat) peptide conjugated quantum dots (TatP-QDs) in complex cellular terrains. Here, TatP-QDs served as nanoscale dynamic pens, which depict remarkable trajectory aggregates of TatP-QDs on the cell surface. Spectral-embedding analysis of the trajectory aggregates revealed a manifold formed by isotropic diffusion and a fraction of directed movement, possibly caused by interaction between the Tat peptides and heparan sulfate groups on the plasma membrane. Further analysis indicated that the membrane deformation induced by Tat-peptide attachment increased with the disruption of the actin framework in cytochalasin D (cyto D)-treated cells, yielding higher interactions on the TatP-QDs. In native cells, the Tat peptides can remodel the actin framework to reduce their interaction with the local membrane environment. Characteristic hot spots for interaction were detected on the membrane, suggesting that a funnel passage may have formed for the Tat-coated particles. This finding offers valuable insight into the cellular delivery of nanoscale cargo, suggesting an avenue for direct therapeutic delivery.

## 1. Introduction

Numerous barriers can be encountered during the delivery of drugs [1], which seriously reduce the efficacy of drugs and increase their off-target toxicity. These barriers include filtration by the kidney and premature clearance by the reticulo-endothelial system. Upon arriving at target tissues, drug molecules must further cross plasma membranes to reach the sites of action. It is particularly desirable to render drug molecules for crossing cellular membranes directly and thereby avoids the complications of vesicle-mediated internalization pathways. Recently, cell-penetrating peptides (CPPs), which are short sequences (8–30) of amino acids (aa) with a net positive charge in water [2], had been found to exhibit the membrane-crossing capability. Since then, CPP-based membrane-translocation strategies have attracted considerable attention, as they can offer a high-delivery yield, low toxicity, and the possibility of tailoring their functions [3]. However, the mechanism by which CPP enters the cell remains unclear [2,3]. An understanding of the cellular uptake of CPP is essential for the development of CPP-based delivery strategies for therapeutic applications in vivo.

In 1988, an 11-aa-long segment (47-57:YGRKKRRQRRR) in the trans activator of transcription (Tat) protein in the human immunodeficiency virus (HIV), also known as the Tat peptide (TatP), was found to display an effective cell-penetrating capability [4]. The interactions involved in the approach of a TatP-coated nanoscale probe may determine whether the uptake of the probe succeeds or fails. Welsher and Yang recently developed a target-locking detection technique for tracking a trajectory of a nanoscale probe in a living cell, revealing a long-range deceleration of the probing particle [5]. However, it remains unclear how long-range forces affect the cell-penetrating capability. The TatP is a prototypical example of an arginine-rich CPP [6]. An in vitro small-angle X-ray scattering study of giant unilamellar vesicles (GUVs) containing actin filaments indicated that arginine-rich peptides are similar enough to GUVs to induce membrane pores with a negative Gaussian curvature [7]. Molecular-dynamics simulations revealed that the free-energy barrier of an arginine-rich peptide moving along a pore path is about 80 kJ/mol lower than that along a pore-free path [8]. TatP can also remodel actin frameworks inside GUVs [9]. A few critical questions remain; for instance, does the mechanism revealed in the in vitro study also occur in living cells? Can cationic CPP interact with negatively charged species on plasma membranes and affect CPP translocation? How do CPPs find the minimum free energy path of translocation near the highly complex and dynamic cellular membrane?

Semiconductor quantum dots (QDs) exhibit the unique optical properties of high emission efficiency, wavelength tunability, and long-term stability, making them appealing as in vivo and in vitro fluorophores [10]. The ability to make QDs water soluble and target them to specific biomolecules has led to promising applications in cellular labelling, sensing and imaging. For example, Zhang et al. developed a QDs-labeled silica nanoprobe for detection of apoptotic cells in response to therapy [11]. Different classes of fluorescent nanoprobes had also been realized for imaging of cellular metal ions [12], which serve as essential cofactors in energy metabolism, signal transduction, and nucleic acid processing. In this study, we take advantage of the brightness and photostability of QDs to investigate the behavior of TatP-coated QDs (TatP-QDs) in living cells at a single-particle resolution. As TatP-QDs translocate across the plasma membranes of living cells, the particles can be viewed as nanoscale pens [5], allowing the influence of the hierarchical structure of the cellular environment on TatP-QD trajectories to be realistically recorded. The interactions involved in the approach of a nanoscale probe may determine whether the uptake of the probe succeeds or fails. Collecting a large number of trajectories and integrating every piece of information on these trajectories may provide a picture of CPP translocation. To this end, we developed a single-particle light-sheet microscopy to capture the translocation of TatP-QDs across the plasma membranes of living cells, which was found to occur preferentially at selected plasma-membrane regions. Analysis of the resulting three-dimensional (3D) trajectories indicated that the interaction between TatP-QDs and heparan sulfate (HS) groups on the plasma membrane [13,14] played a critical role in the observed trajectory aggregation. Upon binding, TatP-QDs can further interact with actin filaments and remodel the actin framework to diminish their interaction with the membrane environment, promoting the translocation of TatP-QDs.

## 2. Materials and Methods

### 2.1. Material Preparation

#### 2.1.1. Preparation of TatP-QD Conjugates

The N terminals of Tat peptides (from Invitrogen, Carlsbad, CA, USA) were biotinylated. Conjugated TatP-QDs were prepared by incubating 20-nm diameter streptavidin-coated QDs (Qdot-585 from Invitrogen) in phosphate buffer saline (PBS) with excess biotinylated TatP (5 μM TatP:50 nM Qdot-585) at room temperature for 30 min. Although streptavidin is a tetramer and each subunit can bind biotin with equal affinity, covalently attached streptavidin to the surface of a quantum dot causes two binding sites to be inaccisible by biotinylated TatP in solution. Therefore, only two effective binding sites are left at each streptavidin [15]. As each QD has approximately 5–10 streptavidin molecules on its surface, we estimated that an average of 14 Tat peptides were conjugated to each QD [16].

The reaction solution was purified with high capacity streptavidin agarose (Thermo Fisher Scientific, Waltham, MA, USA) by filtering out TatP-QDs according to manufacturer’s instructions. To perform single-particle imaging of TatP-QD translocation, a culture medium containing appropriate TatP and TatP-Qdot585 was added to a freshly prepared cell culture at a final concentration of 1 μM TatP and 1 nM TatP-Qdot585. The major species of untagged TatPs were used to rapidly restructure the environment of membrane–peptide interaction, and the minor species of tagged TatP-QDs served as nanoscale dynamic pens, used to trace the landscape of the remodeled membrane–peptide interaction.

#### 2.1.2. Reagents and Cell Culture

HeLa cells were cultured in Dulbecco’s Modified Eagle’s medium with 10% (*v*/*v*) fetal bovine serum without phenol red. Before performing the single-particle tracking, we plated the cells in a slide with eight-well chambers. Once a 70%–80% confluence had been reached, the HeLa cells were deprived of serum for 24 h. To disrupt their actin filaments, the cells were pretreated with a culture medium containing 10 μM cytochalasin D (Cyto D) for 1 h. Fluorescence-activated cell sorting (FACS) showed that when HeLa cells were treated with 10 mU of HS lyase [16] for 2 h at 37 ${}^{\circ }$C, the surface proteoglycans (PGs) can be reduced almost to background levels. Therefore, to achieve a partial removal of surface PGs, we added Heparinase III (Sigma-Aldrich, St. Louis, MO, USA) to a culture medium at a final concentration of 5 mU/mL and incubated the cell culture for 1 h at 37 ${}^{\circ }$C, which reduces the surface PGs to less than 30% of the native value.

### 2.2. Optical Setup

**Figure 1:**
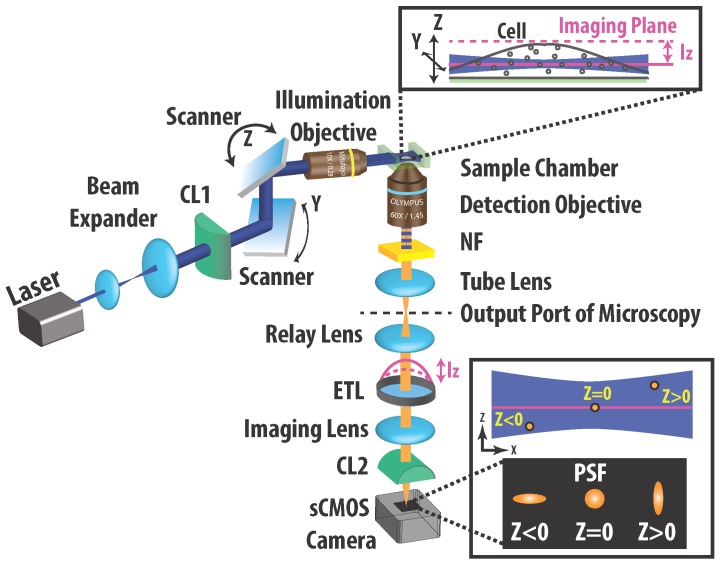
Schematic setup of light sheet microscope used to record 3D trajectories of probing nanoparticles in a living cell. The excitation beam was shaped to form a 3.0 μm-thick light sheet, giving a Rayleigh range of 41 μm. A two-dimensional (2D) scanner was inserted to move the light sheet by 34 μm along the *y*- and *z*-directions at the sample position. The imaging arm was perpendicular to the excitation direction, and an imaging plane in the sample was relayed and imaged to an scientific complementary metal-oxide semiconductor (sCMOS) camera. The position of the imaging plane was adjusted using an electrically tunable lens (ETL) to yield a set of depth-resolved images. An astigmatism was introduced using a CL2 to encode the information on fluorophore depth into an elliptically distorted point spread function.

A schematic diagram of the single-particle light-sheet microscopy apparatus is presented in Figure 1. The output beam from a blue (473 nm) solid-state laser was shaped into a light sheet with a 3 μm beam waist along *z*, which was diffraction-limited to yield a Rayleigh length of 41 μm in the *x*-direction. A scanner was used to move the light sheet by 34 μm along the *y*- and *z*-directions at the sample position with an accuracy of 0.5 μm.

For the imaging, we used a 63 × 1.45 numerical aperture oil immersion objective lens (APON 60XOTIRFM, Olympus Corporation, Center Valley, PA, USA) to ensure both high spatial resolution and high photon collection efficiency. However, the objective lens had a limited depth of field (500 nm). We invoked an astigmatism created by a cylindrical lens (CL2; f = 75 cm) to encode the fluorophore depth as an elliptically distorted point spread function (PSF; see Figure 1), thereby allowing us to localize fluorophores within 1 μm of the imaging focal plane. We further inserted an electrically tunable lens (ETL, Optotune, Dietikon, Switzerland) between the relay and imaging lenses to adjust the position of the imaging focal plane, enabling sharp images at different depths to be acquired rapidly. The fluorescent images of fluorophores in living cells were recorded continuously using a scientific complementary metal-oxide semiconductor (sCMOS) camera (ORCA-Flash 4.0 V2, Hamamatsu Photonics, Shizuoka, Japan).

### 2.3. Localization Accuracy

**Figure 2:**
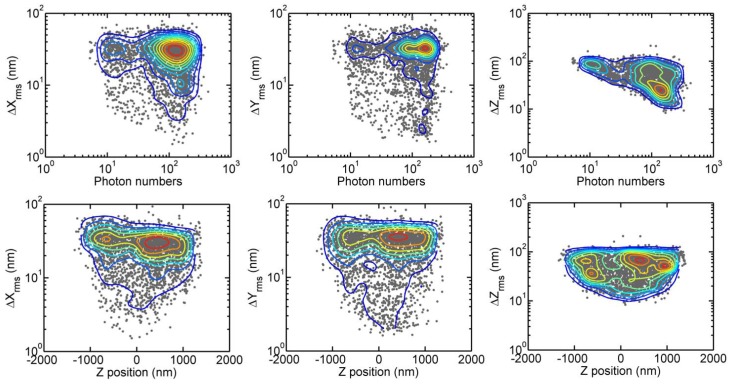
Distribution of localized coordinates of Alexa-488 doped polystyrene beads with a diameter of 100 nm embedded in an agarose gel matrix. (**top row**) Localization errors as a function of fluorescent photon number; (**bottom row**) localization errors as a function of *z*-coordinates of fluorescent beads relative to imaging focal plane (*z* = 0).

A film of 100-nm-diameter fluorescent beads randomly immobilized in 1% agarose gel matrix was prepared to determine the 3D localization accuracy of our apparatus. We took 100 images of the sample with a fixed imaging focal plane and localized the fluorescent beads in a fixed light sheet. We varied the measured signal from *S* = 5 to 400 photons per PSF spot by adjusting the exposure time of the sCMOS camera. This approach yields an estimate of the localization-error function D=(Δx,Δy,Δz,z,S), which was presented in Figure 2. The first row is the plots of *D* on the Δp-*S* plane, where *p* denotes *x*, *y*, or *z*. These plots display our overall localization errors of fluorophores. As shown, we achieve a lateral localization accuracy of 27 nm and an axial localization accuracy of 52 nm at a sufficiently strong signal. The lateral coordinates of the fluorophores can be determined with fairly constant errors for S≃ 8–300 photons. However, the axial localization accuracy is degraded at a weak signal and can become twice as large at S≃8, reflecting the difficulty to determine the shape of elliptically distorted PSF at low photon numbers. The second row shows the Δp-vs-z plots of *D*, confirming the ability to localize fluorophores within 1 μm of the imaging focal plane.

### 2.4. Linking Localized Coordinates of Probing Particles to Generate 3D Trajectories

Single-particle trajectories were recorded for as long as 100 s, with a frame time of 25 ms. The localized coordinates of the fluorophores were extracted from a set of images acquired by synchronously scanning the position of the light sheet and that of the imaging focal plane. Connecting the acquired location coordinates to generate 3D trajectories was challenging. We carried out multiple particle tracking by solving a linear- assignment problem [17] to identify the assignment matrix between the measured location coordinates and their predicted positions with minimal cost. A Kalman filter was also used to provide an optimal estimate for Brownian motion in the presence of Gaussian noise [18]. To verify the functionality of our linking method of 3D-trajectory generation, we simulated a group of particles diffusing in a spatial region with different number densities and diffusion coefficients. The resulting 3D trajectories were coarse-grained with the same sampling scheme as that used with our scanning light-sheet microscope. The simulation results revealed a particle density lower than 0.01 #/μm${}^{2}$, *D* < 0.1 μm${}^{2}$/s, indicating a linking accuracy higher than 99%.

## 3. Results

### 3.1. Uptake of TatP-QDs

**Figure 3:**
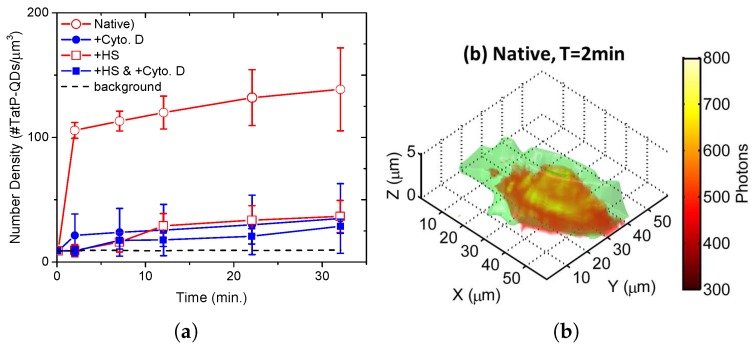
(**a**) time courses of cellular uptake of TatP conjugated quantum dots (TatP-QDs) ($\#/\mu m^{3}$) in native HeLa cells (circles) and heparan sulfate (HS)-removed cells (squares). Data obtained on cells pretreated with Cyto D are presented using filled symbols. Each data point depicts the average value over 10 cells with error bars representing cell-to-cell variation; (**b**) snapshot of spatial distribution of fluorescent signal in a living HeLa cell at 2 min after transactivator of transcription peptide (TatP) administration. Cell surface deduced from optical sectioning phase contrast images is shown in green.

We prepared a cell-culture medium containing 5 μM TatP and 5 nM TatP-QDs to study the initial rates of CPP uptake at room temperature. Figure 3a displays the uptake of TatP-QDs by native HeLa cells (open circles), deduced from time-lapse 3D imaging of TatP-QD measured at various times after the addition of the peptides. Calibration with reference to the brightness of a single QD was used to determine the cellular uptake, which was found to increase rapidly in the first 2 min and then slow down between 2 and 30 min. A snapshot of the spatial distribution of intracellular fluorescence at 2 min is presented in Figure 3b. In the absence of conjugated TatP, we do not observe the bare QDs to associate with the living cells in 30 min at QD concentrations up to 10 nM. However, bare QD uptake by living cells via endocytosis is probable but occurs at longer times than our observation time scale.

The first step in cellular internalization may involve some form of interaction between the cationic peptides and the surface of the cell. The strong anionic charge present on the glycosaminoglycan (GAG) chains of PGs makes them favorable first-binding sites for cationic Tat peptides [14,19,20,21]. To verify the scenario in our case, we treated the cells with Heparinase III enzyme (HSase) to cleave HS groups from heparan sulfate proteoglycans (HSPGs) [19,20]. We observed a reduction in TatP-QD internalization of 74% at 30 min (see open squares in Figure 3a). Treatment with Cyto D, which can inhibit actin polymerization and thereby disrupt the cellular actin framework [22], results in a similar drop (filled symbols) in TatP-QD internalization. The result clearly indicates that both the HS-mediated binding and interaction with intracellular actin filaments are crucial for the initial rapid intake of TatP-QDs.

### 3.2. TatP-QDs Approaching Cell Surface Aggregate at Selected Regions of Plasma Membrane

We prepared a cell-culture medium containing 1 μM TatPs and 1 nM TatP-QDs for single-particle tracking. The major species of untagged TatPs were used to restructure the environment of membrane–peptide interaction, and the tagged species (TatP-QDs) served as nanoscale dynamic pens and payload-carrying vehicles, used to trace out the landscape of the membrane–peptide interaction. We conducted a single-particle trajectory analysis of the TatP-QDs to reveal the underlying dynamics. A unique affordance of our light-sheet microscope was the ability to track TatP-QDs in parallel, providing a global view of the dynamics of the approaching TatP-QDs. However, due to the limited image-taking speed of the camera used, we were only able to track TatP-QDs within a certain distance from the cell surface.

**Figure 4:**
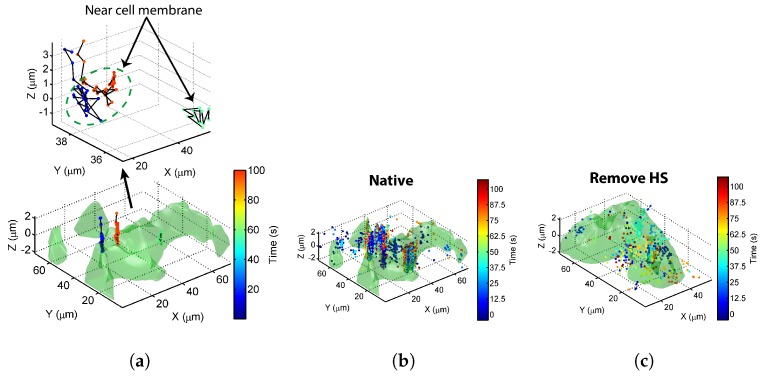
(**a**) three trajectories of TatP-QDs near a living HeLa cell (green) were color-coded to indicate their appearing times. The green profile denotes the cellular surface rendered from optical sectioning phase contrast images; (**b**) when duration was increased to acquire information on more approaching events, trajectory aggregates were observed at selected regions on a native HeLa cell. Each cluster contained up to 150 trajectories; (**c**) time-resolved location coordinates of TatP-QDs near a cell pretreated with HSase to cleave HS groups from membrane-bound HSPGs.

Without external interaction, these Tat-QD nanoscale probes are expected to traverse the extracellular space through a random walk search, attach to the membrane, and then diffuse to find a suitable entrance site. Figure 4a displays three trajectories of TatP-QDs, color-coded to indicate the times of their appearance. The green surface depicts the cell surface rendered from the white light phase contrast images taken by scanning the ETL to position the imaging focal plane at different *z*-coordinates in the living cell. The determination of cell profile is limited by the diffraction effect of the objective lens used, which yields a resolution of 200 nm on the laterial plane and 500 nm in the axial direction. As indicated in the top inset, the initial approaching trajectories of some Tat-QDs resemble directed movement under the force field, and the motion becomes more diffuse as the TatP-QDs come closer to the cell surface. A longer duration of observation to accumulate more approaching events revealed remarkable trajectory aggregates at selected regions of the plasma membrane (Figure 4b). Each cluster contained about 150 trajectories.

The binding affinity of TatP for HSPGs is greater than that for anionic lipids by two to three orders of magnitude. Given that the anionic GAG chains of PGs on the plasma membrane [14,19,20,21] may be favorable binding sites for cationic CPPs, we hypothesized that the trajectory aggregates were caused by HS groups in GAG chains. To verify this conjecture, we treated the cells with HSase to cleave the HS groups from HSPGs. The results are presented in Figure 4c, and show that considerably fewer and more randomly positioned spots were found to be localized in the extracellular space. Spatial analysis of TatP-QD uptake confirmed that the initially rapid uptake correlated with the formation of trajectory clusters. The observed trajectory aggregation seems to have been caused by binding with HS groups in GAG chains on the membranes, suggesting that GAGs play a critical role in redirecting the TatP entry process toward spatially restricted sites on the plasma membrane.

#### Spectral-Embedding Analysis of Trajectory Aggregates of TatP-QDs

As TatP-QDs translocate across the plasma membrane of a living cell, probing particles can record the influences of the cellular environment on their trajectories. We considered the trajectory $\vec{r}\left(t\right)$ to be produced by a stochastic process with a probability density function of $P(\vec{r},t)$, governed by the Fokker–Planck equation [23], which yields a steady-state solution of $P\left(\vec{r},t\to \infty \right)\propto e^{-\int U\left(\vec{r}-\vec{x}\right)\;d\vec{x}}$. This indicates the influence on the probing particle of its local environment. Assuming that the probing particle moves in *n* different realizations of the local environment with corresponding interaction potentials $U_{i}\left(\vec{r}-\vec{x}\right);i=1,..,n$, the mapping from $U_{i}\left(\vec{r}-\vec{x}\right);i=1,..,n$ to $\vec{r}\left(t\right)$ can be imagined as a projection from an *n*-dimensional functional space to a 3D Euclidean space. Recently, Wang and Ferguson used the generalized Takens Delay Embedding Theorem [24] to generate a reconstruction of single-molecule free-energy surfaces from time-series data of a physical observable. To identify the mechanism underlying the trajectory aggregation of TatP-QDs, we focused in this study on retrieving eigenmodes of the translocation of TatP-QDs.

As the structure revealed in $P\left(\vec{r},t\to \infty \right)\propto e^{-\int U\left(\vec{r}-\vec{x}\right)\;d\vec{x}}$ was nonlinear, we first used spectral embedding [25] to extract a low-dimensional manifold from the following set of trajectories: ${\vec{r}}_{i}\left(t\right);i=1,..,n$. A graph-based method provided a useful discretized approximation of the manifold [26] for the efficient construction of eigen-decomposition. The first eigenvector was trivial, and the corresponding eigenvalue gave the data density in a cluster. We then focused on the next two eigenvectors, $\phi_{2}$ and $\phi_{3}$, which offered the most critical information on the interactions between the TatP-QDs and their cellular environments.

**Figure 5:**
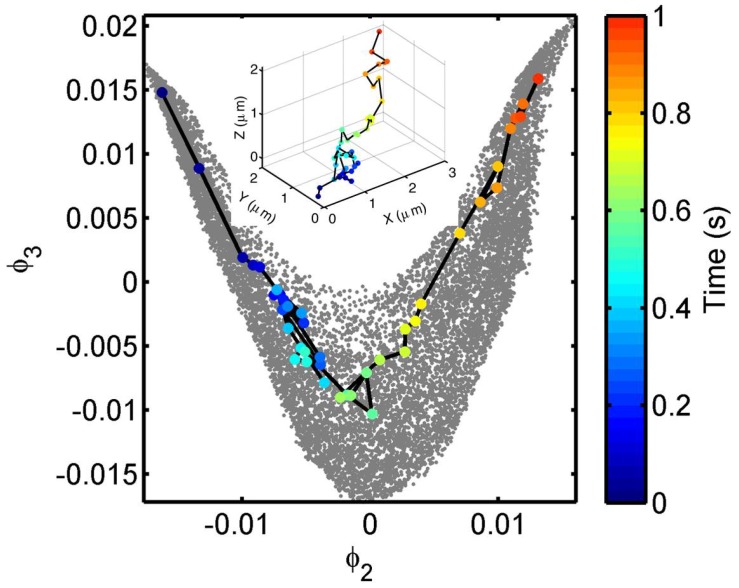
Simulated trajectories (gray) of particle diffusing under a unidirectional force field are presented on a manifold of two principal spectral embedding eigenvectors. A typical trajectory is shown on the manifold and in the real space (inset), with appearing times color-coded.

A particle freely diffusing in a 3D Euclidean space yields an isotropic $P(\vec{r},t\to \infty )$ and therefore a circular distribution in the $\phi_{2}$-$\phi_{3}$ plot. However, the trajectory of a probing particle moving under force field −∇U(r−x) can be produced using the generalized Langevin equation, as follows:

(1) $$m\gamma \partial_{t}r\left(t\right)=-\nabla U\left(r\right)+f,$$

with a frictional parameter *γ* and a thermally fluctuating force *f* satisfying the fluctuation–dissipation theorem $\langle f(t)f(t+\tau )\rangle =2m\gamma k_{B}T\delta \left(\tau \right)$. Solving the equation with $\left|\nabla U\right|/\left|f\right|\;=0.05$ and starting at the origin yields an ensemble of trajectories, we present a typical trajectory in the inset (in real space) and in the $\phi_{2}$-$\phi_{3}$ plot, with appearing time coded by the color template shown to the right of Figure 5. The simulated trajectory data, shown in gray, exhibit a V-shaped distribution in the $\phi_{2}$-$\phi_{3}$ plot, suggesting that the V-shaped feature is a sensitive indicator of directed movement under the force field.

**Figure 6:**
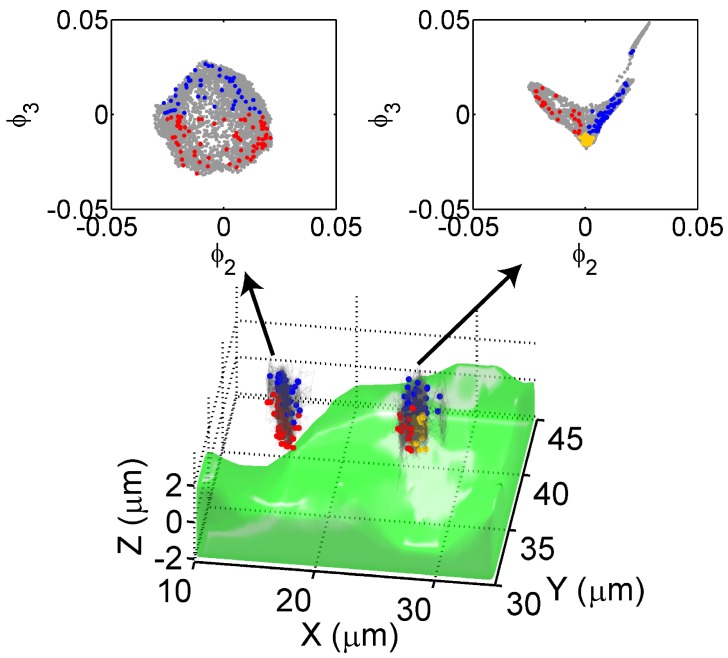
Two trajectory clusters (gray) of TatP-QDs near a living HeLa cell (green) are presented on a manifold of spectral embedding eigenvectors (top inset). For each trajectory cluster, the $\left[V\left(\bar{{R_{\tau }}^{2}}\right)\right]$-$\bar{{\left[R_{\tau }\left(t\right)\right]}^{2}}$ coordinates of the trajectory segments within 2% variance of the peak shown in Figure 7a are displayed in blue on the far side, red near the *z* = 0 plane, and yellow for those closest to the cell membrane.

Figure 6 displays two trajectory aggregates of TatP-QDs: one (left) is near a living cell and the other (right) is directly on top of a cell surface. The green profile denotes the cell surface deduced from the optical sectioning phase contrast method. All of the coordinates of the trajectory aggregates are shown in gray. The location coordinates of TatP-QDs associated with the left cluster present a nearly circular distribution in the $\phi_{2}$-$\phi_{3}$ plot. The right cluster, however, displays a V-shaped distribution. These results indicate that the spectrally decomposed structure of trajectory aggregates carries the information on the interaction of TatP-QDs with their cellular environments.

### 3.3. Influence of Actin Framework on Translocation of TatP-QDs

The findings of a recent study indicated that on attachment to a membrane surface, Tat peptides can remodel the actin framework in an actin-encapsulated GUV [9]. However, it remains unclear whether such multiplexed membrane and cytoskeletal interactions can occur in a living cell. The answer may be found in the trajectories of nanoscale probing particles. To extract relevant stochastic and geometrical structures from the data and gain insights into the mechanism that generated the data, we presented the mean-squared displacement (MSD) $\bar{{\left[R_{\tau }\left(t\right)\right]}^{2}}=\bar{\left[|\vec{r}\left(t+\tau \right)-\vec{r}\left(t\right){|}^{2}+|\vec{r}\left(t\right)-\vec{r}\left(t-\tau \right){|}^{2}\right]/2}$ and normalized variance $V\left(\bar{{R_{\tau }}^{2}}\right)=\sigma^{2}\left(\bar{{R_{\tau }}^{2}}\right)/\bar{{R_{\tau }}^{2}}$ of the trajectories in a contour plot [27,28]. The MSD values were used to quantify the diffusion of a probing particle in its environment, and $V(\bar{{R_{\tau }}^{2}})$ revealed the nature (e.g., deterministic or stochastic) of the interactive forces involved [27]. An attractive feature of this type of plot is that when a molecule repeatedly visits or stays in a spatial region, the characteristics $V(\bar{{R_{\tau }}^{2}})$ and $\bar{{\left[R_{\tau }\left(t\right)\right]}^{2}}$ of the location are imposed on the trajectories, forming a peak at the corresponding position in the plot.

The mathematical foundation of the above method was detailed in our previous publication [27]. Here, we summarize some relevant features of the method to facilitate further study. For a freely diffusive particle, $V(\bar{{R_{\tau }}^{2}})$ has a value of 2. As a particle diffuses under strong confinement, $V(\bar{{R_{\tau }}^{2}})$ can reach a large positive value. This is understandable: as a particle diffuses near a barrier, it may be briefly stalled by the potential, resulting in a large variance in the diffusion step size. In contrast, $V(\bar{{R_{\tau }}^{2}})$ falls below 2 when the probing particle moves in a medium. Due to particle–medium interaction, the probing particle and its nearby ordered medium can be viewed as a dressed particle; and the faster the particle diffuses, the larger the dressing effect is. This results in a smaller variance, and thus a smaller $V(\bar{{R_{\tau }}^{2}})$ for more rapidly diffusing particles.

**Figure 7:**
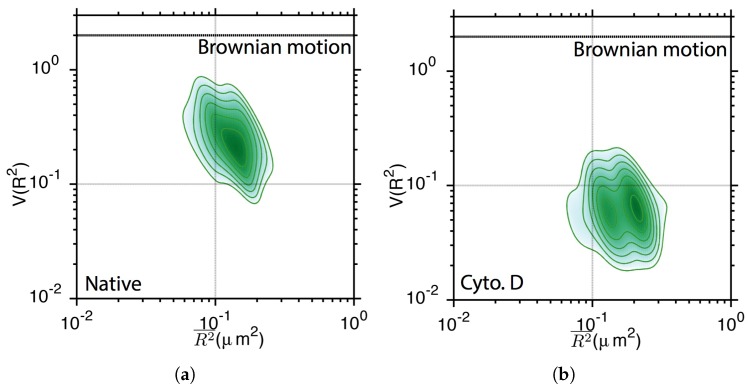
2D contour plot of $\left[V\left(\bar{{R_{\tau }}^{2}}\right)\right]$-$\bar{{\left[R_{\tau }\left(t\right)\right]}^{2}}$ histogram for TatP-QDs moving in the neighborhood of (**a**) living HeLa cell and (**b**) Cyto D-pretreated cell.

Figure 7 displays the $V(\bar{{R_{\tau }}^{2}})$-$\bar{{\left[R_{\tau }\left(t\right)\right]}^{2}}$ contour plot of 23,382 TatP-QD trajectories. A single peak at the coordinates (0.15, 0.21) is shown in Figure 7a, suggesting that the TatP-QDs did not diffuse freely near a native HeLa cell. The peak splits into two and shifts downward to $V(\bar{{R_{\tau }}^{2}})$ = 0.06 for Cyto D-treated cells (see Figure 7b), indicating that TatP-QDs experience stronger interaction with a strained cellular membrane. This finding is understandable, as without the support of an actin framework, the plasma membrane may develop a higher local curvature as a result of TatP-QD attachment. In native cells, the effect of interaction between Tat peptides and the cell membrane may be counter balanced by that of Tat and actin filaments, which results in a higher $V(\bar{{R_{\tau }}^{2}})$.

We analyze each trajectory aggregate by selecting segments that fall within 2% variance of the $V(\bar{{R_{\tau }}^{2}})$-$\bar{{\left[R_{\tau }\left(t\right)\right]}^{2}}$ peak. Labeling the resulting $V(\bar{{R_{\tau }}^{2}})$-$\bar{{\left[R_{\tau }\left(t\right)\right]}^{2}}$ coordinates on the trajectories offers insight into the environmental influences on the TatP-QDs. In the left trajectory cluster, these special points are shown in blue on the far side and red near the *z* = 0 plane. As displayed in Figure 6, the blue dots are uniformly distributed at the rim of the circle in the $\phi_{2}$-$\phi_{3}$ plot, whereas the distribution of red dots, which are close to the cell membrane, appears to be denser on the $\phi_{2}$ > 0 side. In the trajectory aggregate directly on top of the cell, blue dots are located at the right leg ($\phi_{2}$ > 0) and red points dots are concentrated at the left leg ($\phi_{2}$ < 0) of a V-shaped distribution. Yellow dots, which represent trajectory segments closest to the cell membrane, aggregate at the tip of the V-shaped distribution, suggesting the formation of hot spots of interaction on the cell membrane, supported by specifically oriented actin filaments.

### 3.4. Classification of TatP-QD Trajectories

**Figure 8:**
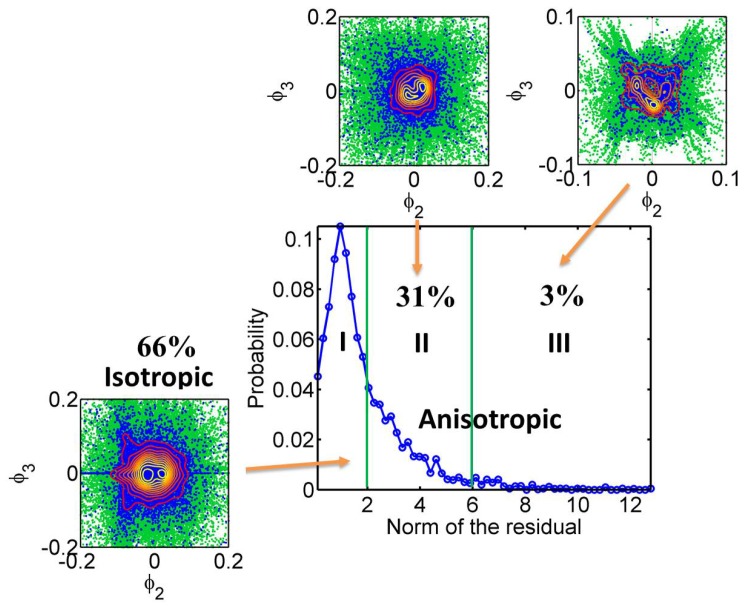
Spectral map (center) and spectral embedding manifold plots (green in insets) of 23,382 trajectories of TatP-QDs measured on 30 living HeLa cells. The $\left[V\left(\bar{{R_{\tau }}^{2}}\right)\right]$-$\bar{{\left[R_{\tau }\left(t\right)\right]}^{2}}$ coordinates of the trajectory segments within 2% variance of the peaks shown in Figure 7 are displayed in blue, with associated contour curves to reveal the peak profiles.

We used spectral embedding to classify 23,382 TatP-QD trajectories measured on 30 cells. The resulting circular or V-shaped distributions in the $\phi_{2}$-$\phi_{3}$ plot are displayed in green in Figure 8. The norm of the residuals, defined as the sum of the squared deviation from the circular distribution of free diffusion, was used as the metric for classification. The coordinates (blue) within 2% variance of the $V(\bar{{R_{\tau }}^{2}})$-$\bar{{\left[R_{\tau }\left(t\right)\right]}^{2}}$ peak and the corresponding contours are also included for comparison. As shown in Figure 8, the class of circular distribution contains about 66% of the data from the native cells, yielding a norm of the residuals of 0.91 ± 0.34. The moderate (2–6) and highly anisotropic (>6) trajectory data occupy 31% and 3%, respectively. We acquired 5112 trajectories for the Cyto D-treated cells. The proportions of the moderate and highly anisotropic trajectories decreased to 27% and 1%, respectively. Treatment with Cyto D reduced the cellular uptake of the TatP-QDs to 25% of that of the native cells, indicating that both the isotropic class and the moderate anisotropic class play a minor role in the initial cellular uptake. The trajectories belonging to the highly anisotropic class resulted in a 75% uptake. These findings may indicate the formation of funnel passages for the TatP-QDs due to the combined effect of HS binding and actin remodeling.

## 4. Discussion and Conclusions

Macchi et al. documented the translocation ability of a specially designed 9-aa peptide fluorophore conjugate, which self-aggregated at selected plasma membrane regions within seconds of peptide administration [29]. A diffuse fluorescence signal was found at μM peptide concentrations, indicating the dominance of a vesicle-independent direct translocation process across the plasma membrane in this concentration regime. Fluorophore-tagged TatPs have also been reported to enter the cytoplasm of living fibroblasts within seconds [30]. The cellular uptake of TatP is highly dependent on the membrane-associated HSPGs [19,20,21]. Dense aggregates bound to membrane-associated HS were found to grow in parallel with the cellular uptake of peptides [28]. The interaction of TatPs with HSPGs may also induce actin organization [19].

Ruan et al. reported that within 5 min of 1-nM TatP-QD addition, the cell outer surfaces were visibly stained [15]. At 30 min, TatP-QDs started to appear inside the cells. These TatP-QDs can be transported to a perinuclear region in an energy-dependent process. Suzuki et al. measured fluorescence resonance energy transfer (FRET) between TatP-QDs (at 30 pM) and membrane-impermeable Alexa546 fluorophore and found that the TatP-QDs remains on the cell surface for at least 20 min. Similar active centripetal movements of TatP-QDs were also observed and this was attributed it HSPG-mediated actomyosin-driven flow [31]. Multivalent TatP-QDs can induce HSPG crosslinking [16]. The resulting TatP-QD and HSPG complex could further trigger membrane actin reorganization that slows down the complex movement [16]. The multivalent TatP-QDs can revisit the same lipid microdomains several times before becoming completely immobilized and entering the cell, suggesting that TatP-QDs that initially interact with HSPGs may cluster on actin-associated lipid microdomains.

It has been proposed that when two oppositely charged biomolecules are brought into contact in a solution phase, a large proportion of the mobile counter ions that previously surrounded each isolated biomolecule are released into the bulk solution [32], which improves the electrostatic interaction between the two oppositely charged biomolecules. When a cationic CPP approaches a membrane comprising anionic species (such as GAG-containing PGs) and neutral lipids, the CPP can polarize the membrane composition by attracting the anionic membrane species to its immediate vicinity. The resulting demixing process can generate a complex interplay between the electrostatic interaction, composition, and curvature elasticity of the membrane [9]. The measured heat capacity change caused by TatP-HS binding in the solution was positive, indicating that the binding was driven mainly by electrostatic forces [14], possibly because the charge density of TatP (+1 e/4.5 Å) and that of HS groups (−1 e/5 Å) are comparable in magnitude but take opposite signs.

Mechanistic insights into the binding-mediated cellular translocation of CPP are critical to the design of successful CPP-based therapeutic delivery systems. We developed a light sheet optical microscope to track TatP-QDs in parallel with high spatial resolution. We focused on the initial step in the translocation of individual TatP-QDs in the complex cellular terrain shortly after peptide administration. To investigate the mechanism underlying the trajectory aggregation of TatP-QDs on living cells, we used spectral embedding to extract an intrinsic low-dimensional manifold from the experimentally measured trajectories. Our result suggests that GAGs play a significant role in redirecting the TatP-QD entry process toward spatially restricted sites on the plasma membrane. The eigenmodes in the direct translocation of TatP-QDs were found to comprise isotropic diffusion and anisotropic directed movements. We used $V(\bar{{R_{\tau }}^{2}})$-$\bar{{\left[R_{\tau }\left(t\right)\right]}^{2}}$ analysis to determine the underlying cause of the trajectory aggregation and found that TatPs may remodel the actin framework to reduce the interaction of TatP-QDs with their cellular environment. Without the support of an actin cytoskeleton, the membrane can be deformed locally by the attachment of TatPs, increasing the interaction between TatP-QDs and the cell membrane. TatPs may exploit these effects to increase the efficiency of membrane translocation. Shortly after peptide administration, TatPs can directly translocate across the plasma membrane by clustering PG receptors and remodeling the actin network. The formation of funnel passages for the TatP-QDs due to the combined effect of HS binding and actin remodeling may be useful for the cellular delivery of macromolecular cargoes.

Recently, the cellular toxicity of unmodified CPPs had been assessed [33], which showed that TatP were essentially harmless at concentrations of up to 100 μM. However, the toxicological properties can be dramatically changed on attachment of cargo. For CPP-conjugated peptidic cargo, the cytotoxicity was found to correlate with the intracellular concentrations of CPP-conjugated cargo, suggesting a possible link between the cytotoxicity of CPP-conjugates and the length of peptidic cargo [33]. The activation of intracellular stress signaling pathways may also be involved. These findings suggest that effector molecules bound to TatP should be designed with the shortest possible sequence and the highest possible affinity for their targets, so that TatP-conjugated cargo concentrations below 10 μM may be applied to cells. Furthermore, differences in cell surface composition are also likely to cause the differences in CPP uptake and onset of toxicity. Addressing the mechanisms of TatP-conjugates induced cytotoxicity was beyond the scope of our present work.

## Supplementary Materials

All of the datasets and software used to support the conclusions of this article are available from the public data repository at the following website: https://zenodo.org/record/59990.

## Abbreviations

The following abbreviations are used in this manuscript:

CPP	Cell-penetrating peptide
cyto D	cytochalasin D
TatP	transactivator of transcription (Tat) peptide
TatP-QD	TatP conjugated quantum dot
HS	heparan sulfate
GAG	glycosaminoglycan
PG	proteoglycan
HSPG	heparan sulfate proteoglycan
PSF	point spread function
ETL	electrically tunable lens
sCMOS	scientific complementary metal-oxide semiconductor
